# Experimental Investigation of the Effect of Steel Fibers on the Flexural Behavior of Corroded Prestressed Reinforced Concrete Beams

**DOI:** 10.3390/ma16041629

**Published:** 2023-02-15

**Authors:** Pouya Hassanvand, Freydoon Rezaie, Mahdi Kioumarsi

**Affiliations:** 1 Department of Civil Engineering, Bu-Ali Sina University, Hamedan 65178-38695, Iran; 2 Department of Built Environment, Oslo Metropolitan University, 0166 Oslo, Norway

**Keywords:** prestressed concrete beam, flexural behavior, fiber reinforced concrete, corrosion, bearing capacity

## Abstract

Rebar corrosion and its consequences are one of the most common damages to reinforced concrete (RC) structures. In structures with greater sensitivity, such as prestressed reinforced concrete (PRC) structures, where steel elements, including prestressed tendons, play a more significant role in supporting the structure, the importance of this issue increases. Methods for repairing and reinforcing such structures have been developed, including incorporating fibers into the concrete mixture to improve its mechanical properties, particularly its bending resistance. This paper presents the results of an experiment that studied the influence of steel fibers on the flexural behavior of PRC beams subjected to accelerated corrosion. Twelve beams with a rectangular cross-section of 150 mm × 300 mm and a length of 2000 mm were fabricated. The steel fibers used in the experiment were corrugated and hooked-end types, with volume fractions of 0.5% and 1.0% in the concrete. Nine beams were subjected to accelerated corrosion testing, with three of them being without fibers and the remaining six being reinforced with steel fibers at volume fractions of 0.5% and 1.0%. Each group of three beams was exposed to three different levels of corrosion, namely 5%, 10%, and 15%. The specimens were tested after exposure to corrosion through a four-point bending load. The accelerated corrosion was induced using an electric current on the prestressing tendons. The results indicated that different levels of corrosion reduced the final bearing capacity and other behavioral characteristics of the specimen, including the amount of energy absorption, effective hardness, and midspan displacement. Adding fibers to the concrete mixture positively affects the compensation of these reduced capacities. Moreover, the amount of this compensation was directly correlated with the volume fraction of used fibers.

## 1. Introduction

Nowadays, reinforced concrete (RC) structures are one of the most common structural systems used worldwide. The high compression strength and the excellent mechanical properties added by the steel made it the ideal compound material to be used in structural applications [1]. Corrosion is recognized as a significant deterioration cause of RC structures [2,3,4]. Indeed, the corrosion attack of reinforcing steel can determine a safety reduction and a change in failure mechanism, even passing from ductile to brittle, and, in the most extreme cases, can cause the collapse of the structure under live loading [5]. Due to the expansion of corroded steel, the corrosion products of a reinforcing bar in concrete exert pressure on the surrounding concrete. This expansion pressure induces tensile stresses in concrete around the reinforcing bar, and the continuous increase in expansion pressure eventually causes cracking through the concrete cover [6,7,8,9]. Corrosion significantly reduces the bearing capacity and ductility of reinforced concrete structures in chloride environments [10,11,12,13,14]. During the service life, the structural performance of corroded structures degrades gradually due to the cracking and spalling of the concrete cover [15], the decrease in the strength of the core concrete [16], the reduction in the cross-sectional area of steel rebar and the deterioration of its mechanical characteristics [17,18], and the decrease in the continuity between concrete and steel rebar [19,20,21]. The potential corrosion risks in prestressed reinforced concrete (PRC) beams would be more severe than in conventional RC beams [22]. Steel prestressing tendons buried within prestressed elements are also vulnerable to corrosion. The corrosion of tendons in prestressed concrete elements can be much more serious than in conventionally reinforced concrete elements since the prestressing tendons have a relatively small cross-sectional area under very high stress [23]. Concrete additives and reinforcing fibers are two new materials that have found a niche in the construction industry. The use of concrete additives improves the desired properties of concrete and compensates for vulnerabilities caused by threats such as corrosion. Fiber materials such as steel, carbon, glass, polypropylene, and nylon in FRC will enhance concrete properties, including tensile strength, stiffness, ductility, crack resistance, fatigue life, durability, impact resistance, and shrinkage reduction capability [24,25,26]. The integration of fiber-reinforced polymer (FRP) rebars with steel rebar in RC beams is another method of improving structural performance. Hybrid steel–FRP-reinforced concrete (HSFRC) beam exhibits ductile failure, and the bearing capacity of the beam continues to increase after yielding [27]. Although the incorporation of steel fiber may result in reduced workability of fresh steel fiber reinforced concrete (SFRC) and in some cases, result in inadequate flowability if the fiber content exceeds a certain threshold [28], it significantly enhances the strength and toughness of the hardened SFRC [29]. Additionally, fibers are used to limit the crack width, which positively affects concrete durability. An increase in the crack width promotes the concrete permeability, favoring the occurrence of corrosion of steel reinforcements [30,31,32].

The mechanisms of concrete failure, such as alkali-aggregate reactions, freeze and thaw, and especially steel corrosion in concrete, have been extensively researched. Zhang et al. [33] investigated the fatigue behavior of corroded PRC beams. It was indicated that fatigue cracks initiate and propagate rapidly around corrosion pits under cyclic loading, and the fatigue failure of prestressing wires at the minimum section where corrosion pits formed was induced by cyclic loading. Moawad et al. [34] studied the behavior of partially prestressed corroded bonded concrete beams. Results indicated that the semi-prestressed concrete beam with the higher compressive strength was more resistant to corrosion than the beam with the lower compressive strength. Ramezanianpour et al. [35] examined rebar corrosion in cracked reinforced concrete under load in the Persian Gulf tidal zone. It is shown that the corrosion of reinforcement is intensified when the ratio of crack width to coating thickness increases. In addition, silica fume reduced the intensity of reinforcement corrosion at the crack location. Taqi et al. [36] conducted an experimental study on the effect of corrosion on the shear strength of fiber-reinforced concrete beams. It is reported that adding fibers improved the shear capacity and reduced the initiation and propagation of cracks due to corrosion, failure mode, and deflection. The failure mode of steel fiber reinforced concrete (SFRC) beams shifted from brittle shear failure to ductile flexural failure.

Corrosion of reinforcements in concrete structures leads to a decrease in their structural performance, and the use of fibers in the concrete mixture improves their mechanical properties. Although many studies have been conducted on the effect of using different fibers on concrete specimens, to the best of the authors’ knowledge, no research exists on the effect of steel fibers on the bending behavior of PRC beams influenced by corrosion. This paper presents an experimental investigation of the effect of steel fibers on flexural behavior in prestressed concrete beams subjected to accelerated corrosion. Twelve prestressed concrete beams were fabricated, nine of which were corroded through an accelerated method using the impressed current. The fibers used were corrugated and hooked-end steel fiber. After exposure to three corrosion levels of 5%, 10%, and 15%, the specimens were subjected to a four-point bending load test. The behavior of the beams was analyzed after conducting a test to record the results and changes caused by corrosion and the impact of steel fibers on their structural performance.

## 2. Experimental Program

### 2.1. Test Specimens

**Figure 1:**
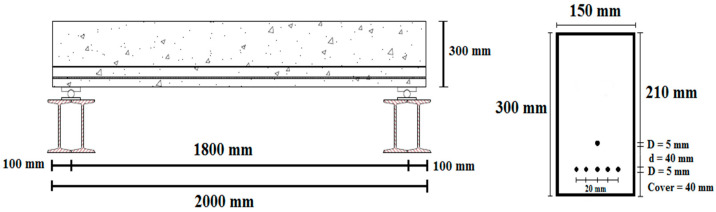
Beam dimensions and cross-section details.

The experimental program consisted of testing 12 pretensioned PRC beams. Each concrete beam was designed and fabricated, with a total length of 2000 mm, a net span of 1800 mm, and a cross-section size of 150 mm × 300 mm. Wire-type prestressing tendons with a 5 mm diameter were utilized to introduce prestress to the specimens. Six prestressing wires were used in the cross-section according to the arrangement shown in Figure 1. Prestressing force was estimated to be approximately 23.7 kN per tendon. However, the loss of tension in prestressed tendons and the loss of jack force were not taken into account. The typical total loss is estimated to be around 20%, resulting in an effective prestressing force of approximately 19 kN. Per Table 20.5.1.3.3 of the ACI 318 [37] standard, the concrete cover over the wire was considered 40 mm. Beam dimensions and cross-section details are shown in Figure 1. Corrugated and hooked-end steel fibers were used in different fractions of 0.5% and 1.0% by volume of concrete. Beams were divided into four groups: A, B, C, and D. Group A served as the reference with beams free of corrosion. Group B contained beams with 5% corrosion, Group C had 10% corrosion, and Group D comprised 15% corrosion. Each group contained one prestressed concrete beam without fibers and two steel fiber-reinforced prestressed concrete (SFRPC) beams with two different fiber volume fractions.

For marking, the specimens without corrosion were designated as C0, those with 5% corrosion as C5, those with 10% corrosion as C10, and those with 15% corrosion as C15. In addition, the NC symbol denotes normal concrete or concrete specimens without fibers, and SF refers to specimens with corrugated and hooked-end steel fiber. The numbers 0.5 and 1 indicate that the fiber content of the specimens is half and one percent of the volume of concrete, respectively. Table 1 presents classification and nomenclature based on the above description.

### 2.2. Material Properties

Coarse (gravel) and fine (sand) aggregates were used to fabricate the specimens. The fine grain used was of the washed broken mountain variety, with a fineness modulus of 2.39, a sand equivalent of 85%, and a specific weight of 25.3 kN/m^3^ in the area under the sieve grade 4 mesh (with an opening diameter of 4.75 mm). The coarse grain used was mountain gravel, with a maximum grain size of 19 mm and a specific weight of 26.1 kN/m^3^. The granulations of used sand and gravel were evaluated per the ASTM C33 standard [38]. In all mixtures, the sand-to-gravel ratio was approximately 0.66. Ordinary Portland cement type (II) with a specific weight of 31.2 kN/m^3^ complied with ASTM C150 standard [39] was used. In addition, a third-generation superplasticizer based on polycarboxylate ether technology with the brand name DEZOBUILD D-40 and a specific weight of 10.4 kN/m^3^ and conforming to the requirements of ASTM C494 standard [40] was used. The manufacturer’s recommendation of 1.0% of the weight of cement in the specimens was followed. 

**Figure 2:**
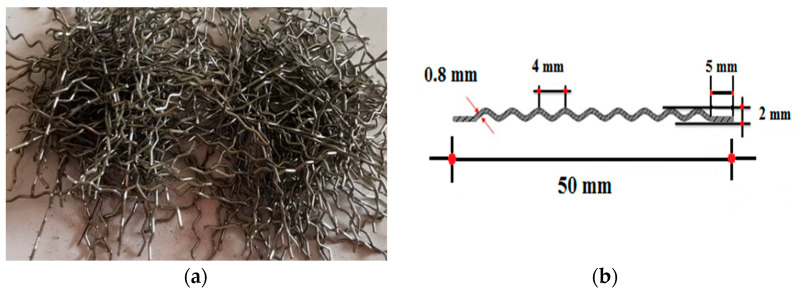
The appearance of corrugated and hooked-end steel fibers: (**a**) Clump of the steel fibersand (**b**) Steel fiber size.

Corrugated and hooked-end steel fibers were utilized in concrete at different fractions by volume of concrete. Figure 2 and Table 2 present the geometric shape and mechanical properties of the steel fibers, respectively.

Wire-type prestressing tendons were utilized to prestress the specimens. In accordance with the pre-tensioned prestressing method, 250-grade and WA-type wires with a 5 mm diameter were used per the American ASTM A421 standard [41]. High-strength steel was used to manufacture the wires. The tensile strength of the employed wires was 1725 MPa. The mechanical properties of prestressing wires were tested, and the results are given in Table 3.

### 2.3. Mix Design

The concrete used in the beams was supplied by one of the prestressed concrete joist manufacturers with a long-line system. In order to achieve the selected target compressive strength, several concrete mixing designs were investigated, and a design for making concrete specimens was chosen through the weight method, according to Table 4. The water-to-cement ratio (W/C) was kept constant and equaled 0.38 for each test specimen. The compressive strength of the concrete without fibers, measured at room temperature after 28 days, was recorded as 38.8 MPa. Meanwhile, the tensile strength of the concrete without fibers was determined to be 3.9 MPa.

### 2.4. Casting and Curing of Prestressed Concrete Beams

**Figure 3:**
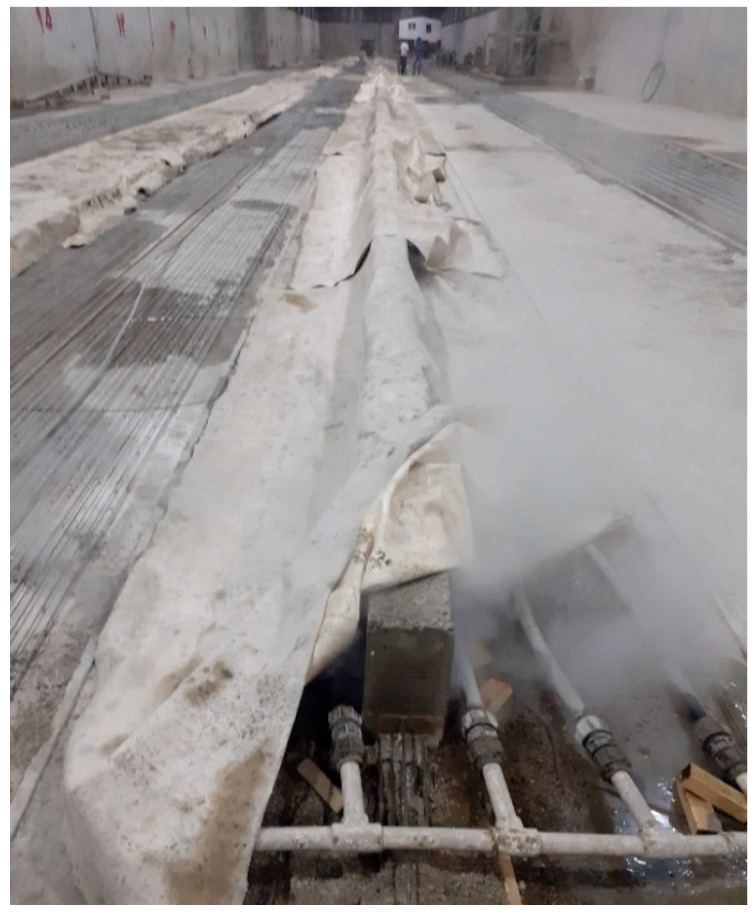
Curing of specimens via the steam method.

The concrete was mixed using a batching plant, which ensured accurate measurements of the weight of cement, sand, and gravel, as well as water, through the use of high-precision digital measuring equipment. The test specimens were molded using metal molds. Before pouring the concrete, the reinforcing wires were restrained at both ends using mechanical anchorage. Each prestressing wire was pretensioned up to 70% of its nominal ultimate tensile strength (F_pu_), equivalent to about 23.7 kN per wire. After the concrete was poured and allowed to set for 48 h, the side metal molds were removed. Then, the beams were cured using the steam curing method. In this manner, following the production of the beams, a set known as a steam blanket was applied to the beams per the images, see Figure 3. This system was outfitted with a grid of porous steam pipes. The temperature of the produced steam gradually reached 70 °C and the beams remained at this temperature for a certain period while under the steam blanket. The temperature then decreased and gradually approached a cool state. The cured products were identical and uniform along the length of the beam, and they were ready for loading and operation after 24 h.

### 2.5. Accelerated Corrosion Process

**Figure 4:**
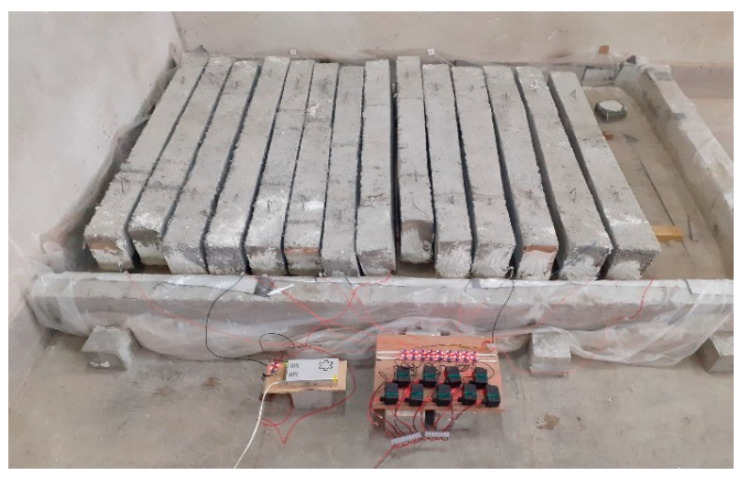
Accelerated corrosion of the prestressed concrete beams.

**Figure 5:**
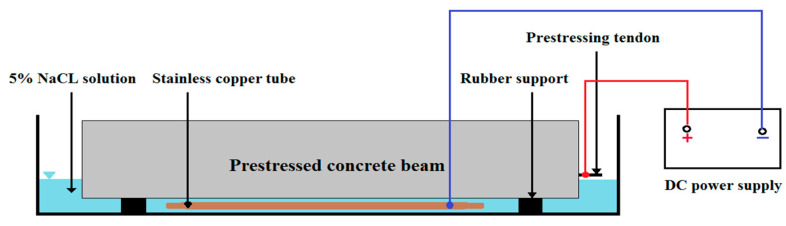
Schematic view of the corrosion test setup.

The corrosion of the specimens was accelerated through the application of an electric current. Electrochemical action and the application of a constant current intensity between the anode (prestressing wires) and the stainless tube (copper tube) as the cathode outside the specimen accelerated corrosion. The corrosion process was initiated by placing the specimens and the cathode in a pool containing a 5% sodium chloride solution, as present in Figure 4, and establishing a constant electric current between the anode and the cathode using a power supply with a constant electric current. The solution level was enough to submerge the prestressing wires. The applied current density was maintained at 150 µA/cm^2^, and the power supply voltage was kept below 12 volts. As a comparison, the current density in most recent studies is commonly chosen in the range of 100 to 500 µA/cm^2^ [42]. Figure 5 illustrates a schematic representation of the laboratory’s electrical circuit used to accelerate corrosion. The accelerated corrosion method is based on the method presented in Zhang et al. [33] research. Different durations of the impressed current were utilized per Faraday’s law to achieve varying degrees of corrosion of the wires in the beams. The duration of applying electric current to the specimens in order to cause accelerated corrosion at different levels and the actual corrosion rate are presented in Table 5.

**Figure 6:**
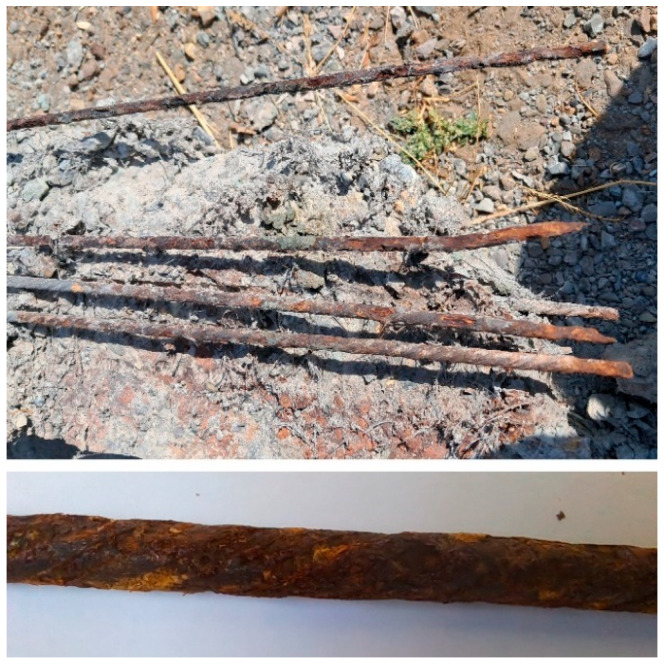
Corroded prestressing wires.

**Figure 7:**
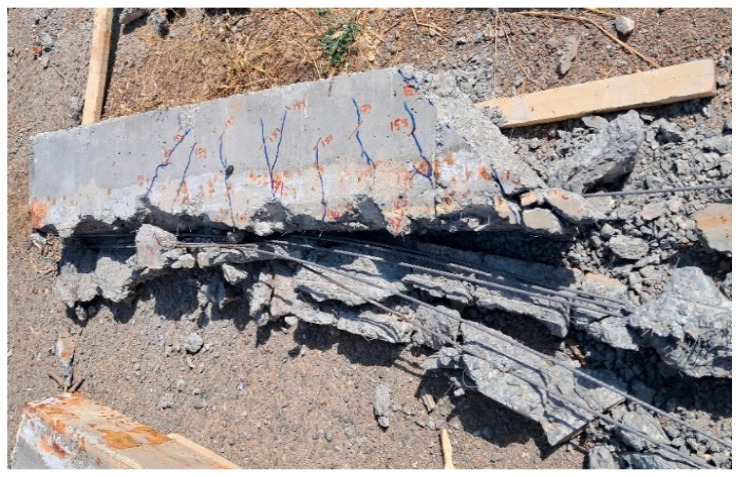
Destruction of specimens and removal of prestressing wires.

Figure 6 shows an example of corroded prestressing wires after destroying the concrete and removing them, as shown in Figure 7.

### 2.6. Test Setup and Instrumentation

**Figure 8:**
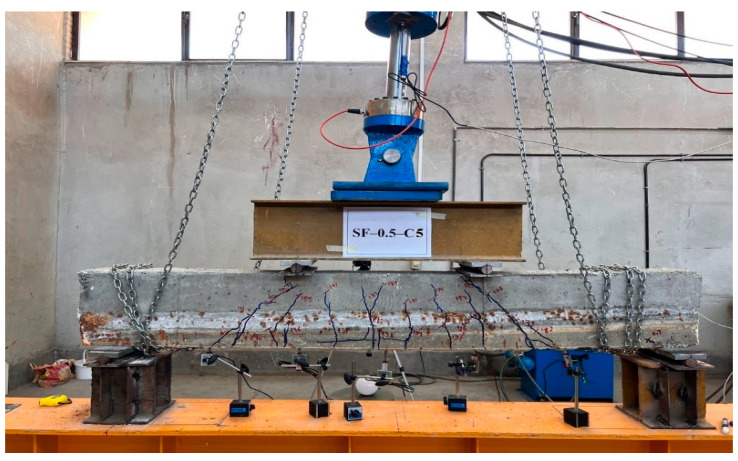
Four-point bending test setup.

**Figure 9:**
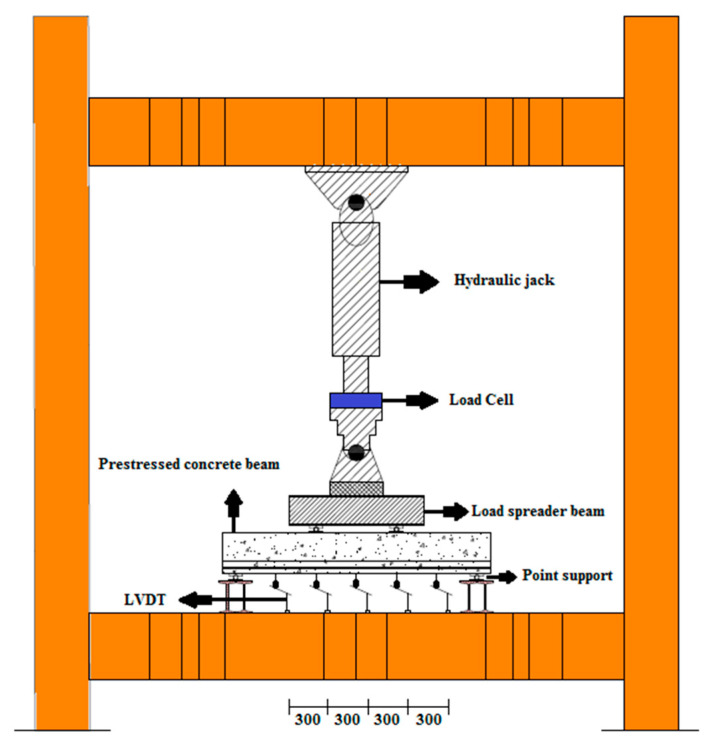
Schematic of the four-point flexural test setup.

All the beams were tested in a four-point bending configuration. Loading was applied monotonically in displacement control mode at a rate of 0.01 mm/s. Load from the actuator was transferred to the specimen through the spreader beam and then to the double I-beams. A photograph of the four-point bending test setup is shown in Figure 8. Loading continued until the specimens completely failed. The ultimate failure of the specimens resulted from the degradation of the bond between the concrete and reinforcement caused by corrosion, which gave rise to the formation of cracking patterns. Specifically, bending-shear and bending cracks were observed to develop beneath the beam and propagate toward the beam’s top edge. These cracks ultimately led to the complete failure of the specimens. Linear variable displacement transducers (LVDT) were used to measure the vertical displacement at various beam points; a load cell measured the amount of load, and a hydraulic jack and pump were used to apply the load. The capacity of the jack used in this experiment was 500 kN. The LVDTs were positioned at specific locations (at distances of one-sixth of the net span) along the length of the beam to capture the entire curvature profile during testing. The cracks that formed and their growth paths in the beams were documented by marking them on the surface of the specimens. In addition, the force responsible for each crack was also recorded. The beam schematic and loading configuration are presented in Figure 9.

## 3. Test Results and Discussions

### 3.1. Load–Midspan Displacement Behavior

The experimental load–midspan displacement diagrams, which were used to demonstrate the structural behavior of beams based on the method proposed by Paulay and Priestley [43], were idealized and converted into an equivalent bilinear diagram. The seismic characteristics of the beams, including effective hardness and ductility, can be calculated using this equalized diagram. The equivalent bilinear diagram was generated by equalizing the enclosed area between the load–displacement curve and the diagram. This was completed by ensuring that the area under both curves was the same. In addition, the structure’s stiffness after yielding (secondary slope of the bilinear curve) is zero. Therefore, effective hardness equals the hardness of the specimen’s yield point in the load–midspan displacement diagram. In other words, the effective hardness, which according to the definition is the amount of an object’s resistance to deformation, is equal to the slope of the load–midspan displacement diagram line that connects the origin to the point at which 75% of the maximum load is applied on the load–midspan displacement diagram [43].

**Figure 10:**
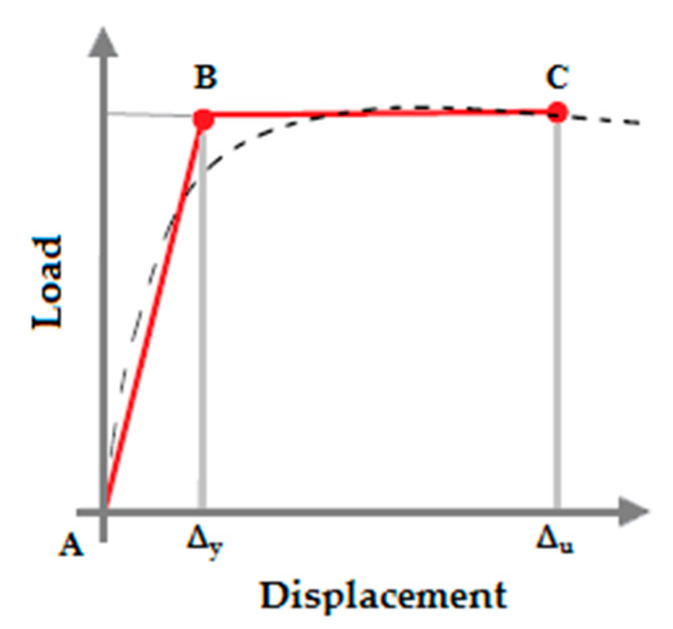
Idealization of the load–displacement curve with an equivalent bilinear diagram.

The ductility µ, which is defined as the ability to withstand hyperelastic deformations in a part or the entire length of a structural element without appreciable resistance loss, is derived from the equivalent bilinear diagram by calculating the ratio of the final displacement ∆_u_ to the yield displacement ∆_y_ (Equation (1)). The final and yield displacements were calculated using the equivalent bilinear diagram. The yield displacement corresponds to the endpoint of the first linear segment and the beginning of the second linear segment, as depicted in Figure 10. The endpoint of the second linear segment in the equivalent bilinear diagram represents the final displacement.

(1) $$µ=\frac{\Delta u}{\Delta y}$$

**Figure 11:**
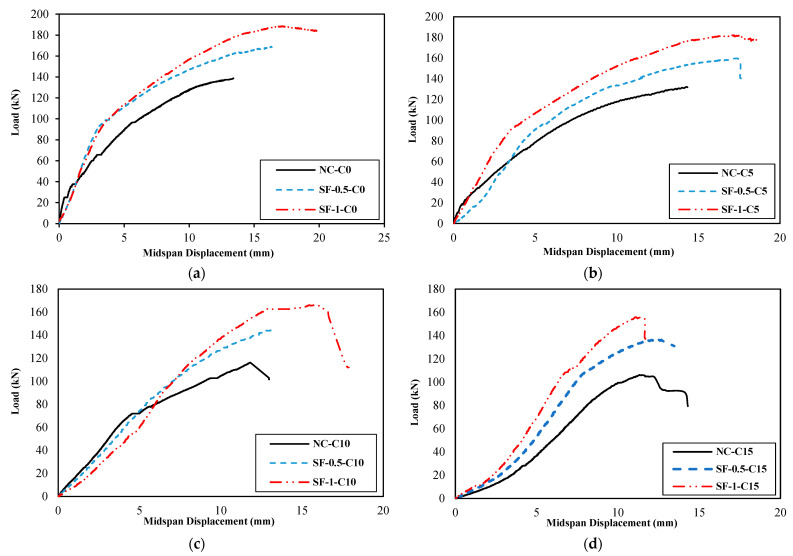
Load–midspan displacement diagrams: (**a**) Group A specimens, (**b**) Group B specimens, (**c**) Group C specimens, and (**d**) Group D specimens.

**Figure 12:**
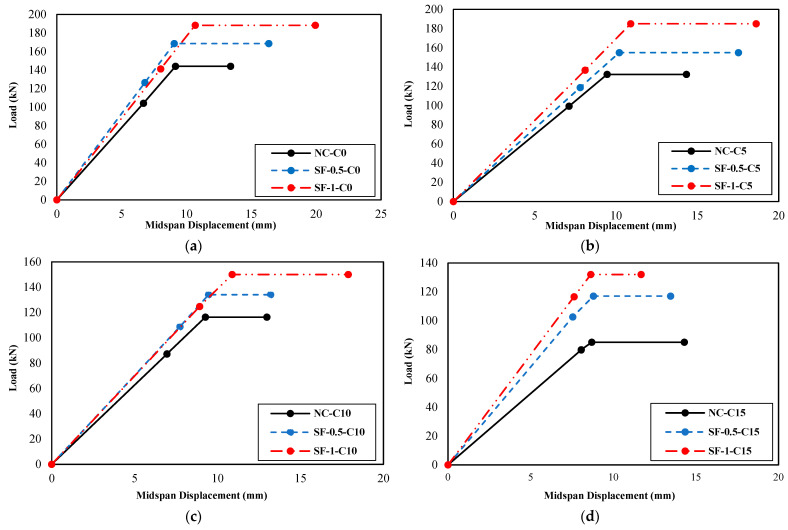
Equivalent bilinear diagrams: (**a**) Group A specimens, (**b**) Group B specimens, (**c**) Group C specimens, and (**d**) Group D specimens.

Figure 11 presents the load–midspan displacement diagrams of groups A, B, C, and D, and Figure 12 illustrates their equivalent bilinear diagrams for comparison purposes.

As shown in the diagrams of Figure 11 and Figure 12 and the results presented in Table 6, it can conclude that the steel fiber positively affected the bearing capacity, energy absorption, and final displacement of the beams.

As calculated theoretically, the expected moment capacity for the NC-C0 beam was 41.7 kN·m, and the measured moment capacity in the laboratory was 42.1 kN·m. A minor discrepancy of approximately 1% between the theoretical and actual values may have arisen from errors during the specimen fabrication.

Regarding the bearing capacity of the PRC beams, corrosion at different levels caused a reduction. At the corrosion level of 15%, corrosion reduced the bearing capacity of the beam NC-C0 by approximately 24%. The reduction of bearing capacity caused by corrosion and its intensification in increasing the level of corrosion were also presented in the literature [10,18,22,36,44]. On the other hand, the SFRPC beams, compared to the PRC beam without fibers, increased the bearing capacity and based on the area under the diagram, increased its energy absorption in a state without corrosion. Furthermore, the SF-0.5-C5 beam, compared to the NC-C5 beam, compensated for the bearing capacity loss caused by corrosion at 5%. The SF-0.5-C10 beam, compared to the NC-C10 beam, compensated for the bearing capacity loss caused by corrosion at 10%. The SF-0.5-C15 beam, compared to the NC-C15 beam, compensated 94% of the reduction in bearing capacity caused by corrosion at 15%. The SF-1-C5, SF-1-C10, and SF-1-C15 beams, compared to the NC-C5, NC-C10, and NC-C15 beams, compensated and increased the lost bearing capacity at the corrosion levels of 5%, 10%, and 15%. 

The loading was performed in this study by four-point bending, and as a result, the bending capacity could be calculated based on the bearing capacity. The impact of the fibers on the bending capacity is equivalent to its impact on the bearing capacity. Previous research studies such as [10,16,22] have shown that corrosion decreases the bending capacity of the specimens. By incorporating steel fibers into the concrete mixture, the prestressed concrete beams perform better both in the presence and absence of corrosion. Moreover, specimens with a fiber volume fraction of 0.5% and 1.0% were able to compensate for the bending capacity reduction caused by corrosion compared to specimens without fibers. Corrosion caused a reduction in the energy absorption of the beam NC-C0 at different levels, with a 34% reduction at the corrosion level of 15%. In the SF-0.5-C5 beam compared to the NC-C5 beam, the decrease in energy absorption capacity caused by corrosion at a level of 5% is compensated and increased. The SF-0.5-C10 and SF-0.5-C15 beams compensated for 51% and 45% of the reduction in energy absorption caused by corrosion at levels 10% and 15% compared to the NC-C10, and NC-C15 beams, respectively. The SF-1-C5 and SF-1-C10 beams, compared to the NC-C5 and NC-C10 beams, respectively, compensated and increased the lost energy absorption at the corrosion levels of 5% and 10%. The SF-1-C15 beam compensated for 27% of the reduction in energy absorption caused by corrosion at level 15%.

The 5% increase in corrosion level resulted in a 7% increase in the final displacement of the beam. However, a 10% corrosion level resulted in a decrease of 12% in the final displacement of the beam. The most significant decrease of 19% in the final displacement was observed in the NC-C0 beam with a corrosion level of 15%. The SF-0.5-C5 beam improved the final displacement by approximately 24% at a corrosion level of 5% compared to the NC-C5 beam. Moreover, it was observed that the SF-0.5-C5 beam exhibited an increase in final displacement of approximately 24% compared to the NC-C5 beam when the corrosion level was 5%. The SF-0.5-C10 and SF-0.5-C15 beams were observed to compensate for a substantial proportion of the decrease in final displacement caused by the corrosion levels of 10% and 15%. The compensation rate was found to be 88% and 48%, respectively, as compared to the NC-C10 and NC-C15 beams. An examination of the final displacement of the SF-1-C5 beam, in comparison to the NC-C5 beam, indicated an increase of approximately 20% at a corrosion level of 5%. The SF-1-C10 beam, in comparison to the NC-C10 beam, fully compensated for the decrease in final displacement at a corrosion level of 10%. The SF-1-C15 beam, compared to the NC-C15 beam, partially compensated for the reduction in final displacement resulting from corrosion at a level of 15% with a compensation rate of 16%. The positive effect of steel fibers on the performance of corroded concrete beams has been shown in the research results of Faten Y. et al. [36].

Corrosion at levels 5% and 15% led to an increase in ductility, while corrosion at level 10% resulted in a 4% decrease in ductility compared to the reference beam (NC-C0). The SF-0.5-C5 beam, compared to the NC-C5 beam, increased the ductility by approximately 14% at the corrosion level of 5%. Steel fibers did not affect the ductility of the NC-C10 beam at the corrosion level of 10%. The SF-0.5-C15 beam decreased the ductility at the corrosion level of 15% by 8%, compared to the NC-C15 beam. The SF-1-C5 beam increased the ductility by approximately 14% compared to the NC-C5 beam at the corrosion level of 5%. The SF-1-C10 beam completely compensated for the lost ductility at the corrosion level of 10%, compared to the NC-C10 beam. The SF-1-C15 beam compensated for 18% of the reduction in ductility caused by corrosion at level 15%, compared to the NC-C15 beam.

The most significant decrease in the effective hardness, around 37%, was observed in the beam with a corrosion level of 15%. The SF-0.5-C5, SF-0.5-C10, and SF-0.5-C15 beams compensated 77%, 50%, and 65% of the lost effective hardness at the corrosion levels of 5%, 10%, and 15%, respectively, compared to the NC-C5, NC-C10, and NC-C15 beams. The SF-1-C5 beam completely compensated for the lost effective hardness at the corrosion level of 5% compared to the NC-C5 beam. The SF-1-C10 and SF-1-C15 beams compensated for 47% and 95% of the lost effective hardness at corrosion levels of 10% and 15%, respectively, compared to the NC-C10 and NC-C15 beams.

**Figure 13:**
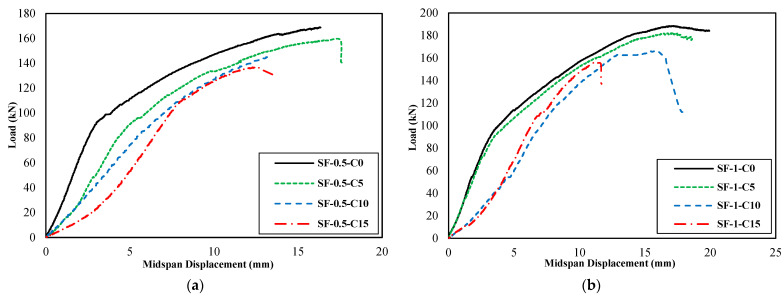
Load–midspan displacement diagrams: (**a**) specimens with a 0.5% volume fraction, (**b**) specimens with a 1.0% volume fraction.

Figure 13 displays the comparative diagram of specimens with the same volume fraction of steel fibers subjected to different levels of corrosion.

As seen in the diagram in Figure 13, with the increase in corrosion levels, the structural performance parameters, including bearing capacity, energy absorption rate, and final displacement, decrease both in specimens with a 0.5% volume fraction and those with a 1.0% volume fraction.

**Figure 14:**
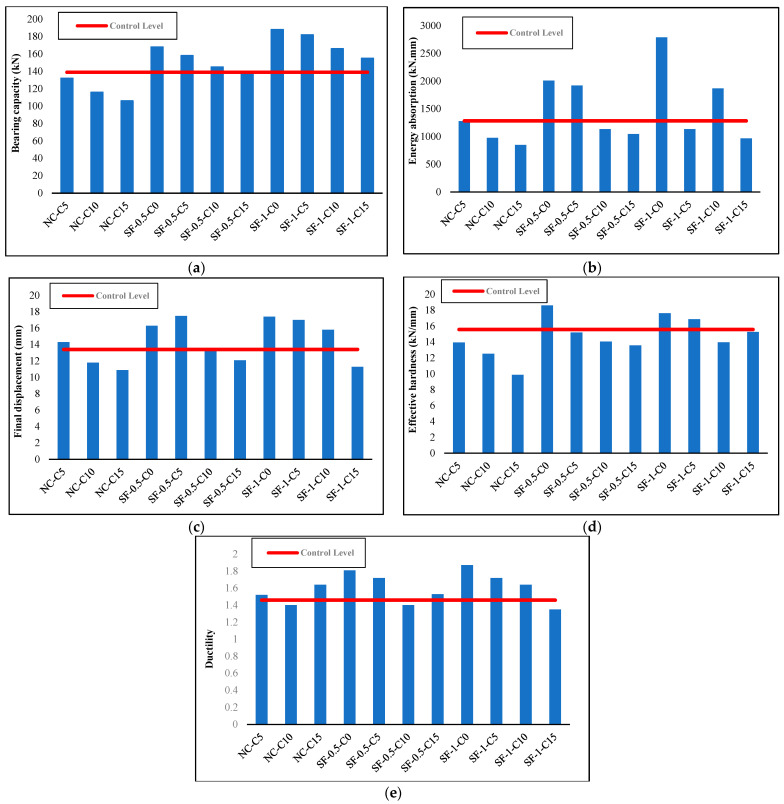
Comparative diagram of the change in (**a**) bearing capacity, (**b**) energy absorption, (**c**) final displacement, (**d**) effective hardness, and (**e**) ductility of the beams compared to the control specimen.

Comparative diagrams of the investigated parameters for the structural performance are presented in Figure 14.

**Figure 15:**
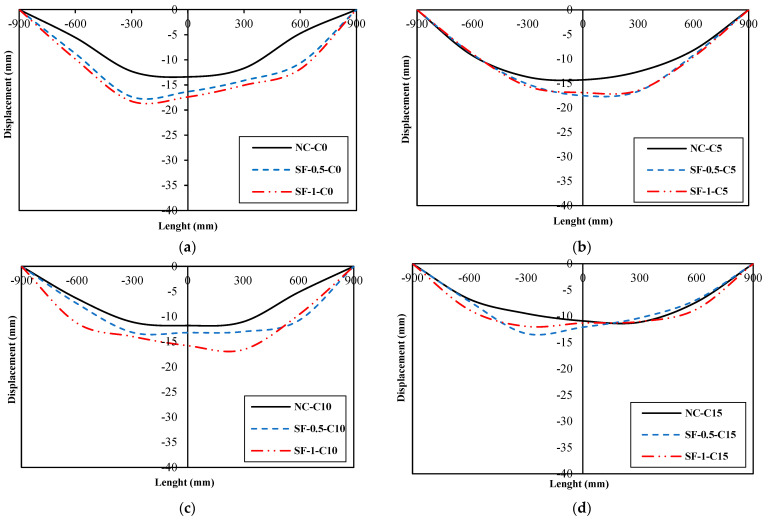
Displacement longitudinal profile diagrams: (**a**) Group A specimens (**b**) Group B specimens (**c**) Group C specimens (**d**) Group D specimens.

Figure 15 presents the displacement longitudinal profile diagrams of group A, B, C, and D specimens. The longitudinal displacement profile of the beams is drawn at a load equivalent to 90% of the bearing capacity.

Displacement longitudinal profile diagrams of all beams are asymmetric, and the value of displacement in the half of the failing beam is greater than in the other half. 

Table 6 summarizes the results of the tests conducted on four groups of beams A, B, C, and D for this study.

### 3.2. Comparison of the Effect of Changes in Corrosion Levels on the Structural Behavior Characteristics of the Specimens

**Figure 16:**
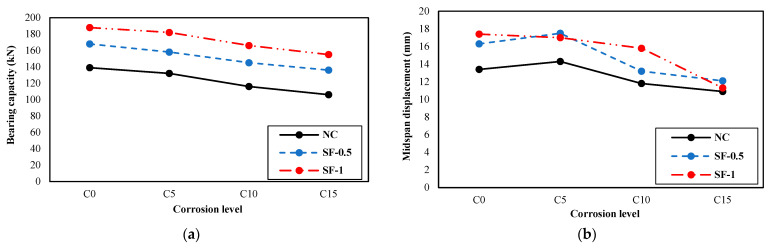

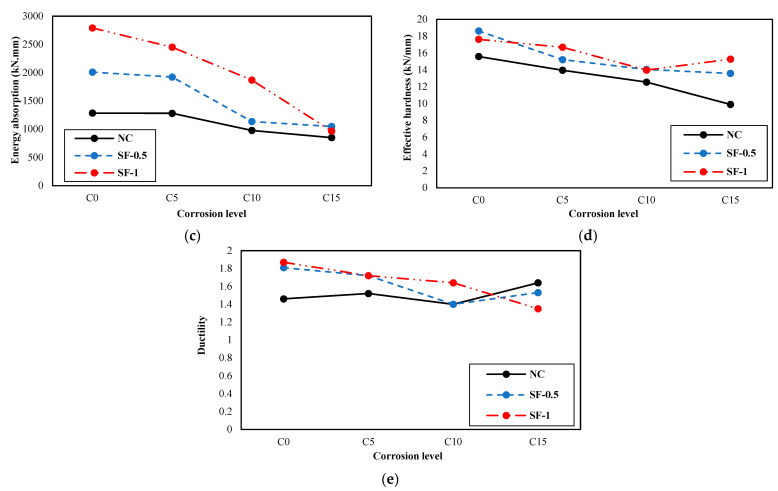
Changes in the studied characteristics with increasing corrosion levels: (**a**) bearing capacity, (**b**) midspan displacement, (**c**) energy absorption, (**d**) effective hardness, and (**e**) ductility.

Figure 16 depicts the trend diagrams of the changes in the studied characteristics in the flexural behavior of the beam under the influence of corrosion at different levels.

The diagrams show that the values of the investigated parameters for the bending behavior of the beam decreased with increasing corrosion levels for most of the specimens. The largest reductions in most beams occurred from 5% to 10% corrosion. However, the investigated structural behavior parameters increased in the specimens with steel fibers. There is a direct correlation between the increase in these values and the volume fraction of fibers used.

### 3.3. Failure Mode and Crack Propagation Pattern in Specimens

**Figure 17:**
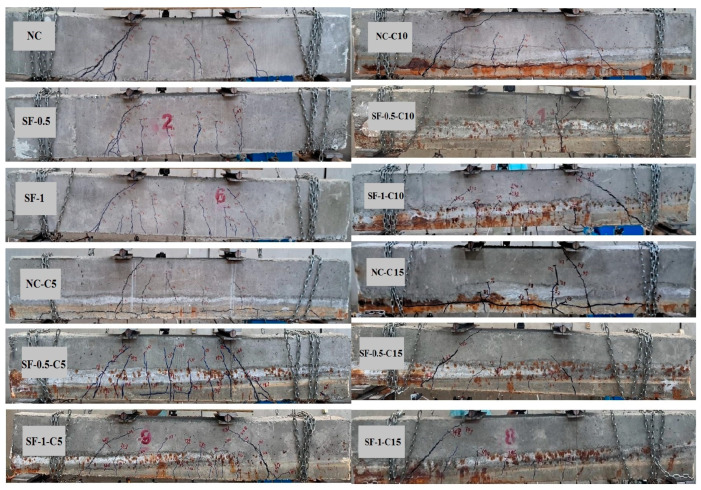
Failure mode and crack pattern of beams.

The failure mode and crack propagation of the beams are shown in Figure 17. The addition of steel fibers altered the failure mode of the two beams (SF-0.5-C0 and SF-1-C0) from shear failure to flexure-shear for the beams without corrosion. At the corrosion level of 5% for beams with steel fibers, it was observed that some shear cracks appeared in the region between the supports and loads, in addition to some short flexural cracks appearing in the bending region, as shown in Figure 17. It was observed that the corrosion level of 15% significantly impacted the failure mode of the beams (NC-C15, SF-0.5-C15, and SF-1-C15), causing them to fail in a brittle manner. This is because severe localized corrosion was found in the extracted prestressing wires after the failure test. It was observed that different levels of corrosion reduce bond strength and cause continuity crack at the region of the wires and sudden shear and splitting for beams without steel fibers (NC-C5, NC-C10, and NC-C15), as shown in Figure 17. The research results presented in [16,20] also indicate a decrease in bond strength due to corrosion. It can also be concluded that the presence of steel fibers decreases the width of cracks.

## 4. Conclusions

In this study, twelve prestressed concrete beams were fabricated, with nine subjected to accelerated corrosion by electric current. The research aimed to examine the flexural behavior of these beams under the influence of corrosion and the effect of corrugated and hooked-end steel fibers on structural performance. The key findings based on laboratory results were:

The correlation between the theoretical predictions based on Faraday’s law and actual corrosion was found to have a difference of approximately 1–3% for varying levels of corrosion.

- The study found that tendons near the sidewalls of the beams experienced more corrosion compared to the internal wires. Furthermore, the corrosion rate was higher at the beginning and end of the tendons than in the middle sections.
- SFRPC beams with 0.5% or 1.0% fiber volume fraction improved bearing capacity at corrosion levels of 5%, 10%, and 15% compared to the PRC beam without fibers.
- The bearing capacity increased with fiber volume fraction.
- Steel fibers positively impacted the beams’ energy absorption, effective stiffness, and ductility at different corrosion levels.
- The final displacement of the beams decreases as the corrosion level increases, both for corroded and uncorroded beams.
- Displacement of specimens with fibers was more remarkable than without fibers, and the cracking load decreased as corrosion levels increased but was compensated by using fibers.
- Bending cracks initiated near the midspan of beams and extended at the height of the tension zone, with shear cracks forming close to support.
- As corrosion levels increased, the cracking load ratio decreased but was compensated by SFRPC beams with different volume fractions compared to the PRC beam without fibers.

This study has focused on the impact of corrosion on prestressing tendons, however, the effect of corrosion on steel fibers has not been considered. Future investigations may explore the combined effect of corrosion on both steel fibers and tendons on the performance of prestressed reinforced concrete beams and examine the feasibility of using alternative fibers.

## Figures and Tables

**Table 1:** Specimens classification and marking specimens based on corrosion level and fiber volume fractions.

Group	Specimens Mark	Dimensions (mm)	Corrosion Percentage	Type of Fiber	Volume Fraction of Concrete	Prestressing Force per Each Wire
						(kN)
A	NC-C0	2000 × 300 × 150	Control specimen	No fibers	0	23.7
	SF-0.5-C0			Steel	0.50%	23.7
	SF-1-C0			Steel	1%	23.7
B	NC-C5	2000 × 300 × 150	5	No fibers	0	23.7
	SF-0.5-C5			Steel	0.50%	23.7
	SF-1-C5			Steel	1%	23.7
C	NC-C10	2000 × 300 × 150	10	No fibers	0	23.7
	SF-0.5-C10			Steel	0.50%	23.7
	SF-1-C10			Steel	1%	23.7
D	NC-C15	2000 × 300 × 150	15	No fibers	0	23.7
	SF-0.5-C15			Steel	0.50%	23.7
	SF-1-C15			Steel	1%	23.7

**Table 2:** Mechanical properties of steel fibers.

Primary Material	Specific Weight (kN/m^3^)	Fiber Length (mm)	Diameter (mm)	Aspect Ratio	Tensile Strength (MPa)	Percentage Increase in Length	Modulus of Elasticity (MPa)
Low-carbon and cold-rolled steel	78.5	50 mm	0.8	62.5	700	3%	200,000

**Table 3:** Mechanical properties of prestressing wires.

Diameter (mm)	Cross-Section (mm^2^)	Weight per Unit Length (kN/m)	Modulus of Elasticity (MPa)	Tensile Strength (MPa)	Minimum Elongation
5	19.6	0.00153	1.965 × 10^5^	1725	3.5% for a length of 600 mm

**Table 4:** Mixing design details (in kg/m^3^).

Type	Gravel	Sand	Cement	Water	W/C	Steel Fiber	Superplasticizer
NC	1065	710	450	171	0.38	-	4.5
SF-0.5	1045	690	450	171	0.38	39.25	4.5
SF-1	1020	675	450	171	0.38	78.5	4.5

**Table 5:** Corrosion time and actual corrosion rate for the accelerated corrosion process.

Corrosion Level	Corrosion Time (h)	Corrosion per Faraday’s Law (%)	Actual Corrosion (%)
One	491	5	3.85
Two	982	10	8.68
Three	1473	15	12.47

**Table 6:** Summary of the test results.

Group	Beam Mark	Cracking Load P_cr_ (kN)	Midspan Displacement Corresponding to Cracking Load Δ_cr_ (mm)	Bearing Capacity P_u_ (kN)	Midspan Displacement Corresponding to the Bearing Capacity Δ_u_ (mm)	The Ratio of Cracking Load to Bearing Capacity P_cr_/P_u_	Ductility µ	Effective Hardness K_eff_ (kN/mm)	Absorbing Energy E (kN·mm)
A	NC-C0	89	4.97	139	13.4	0.64	1.46	15.58	1284
	SF-0.5-C0	105	4.32	168	16.3	0.625	1.81	18.62	2009
	SF-1-C0	115	5.22	188	17.4	0.611	1.87	17.62	2791
B	NC-C5	75	4.55	132	14.3	0.568	1.52	13.95	1280
	SF-0.5-C5	102	5.52	158	17.5	0.645	1.72	15.21	1923
	SF-1-C5	100	4.37	182	17	0.549	1.72	16.88	2451
C	NC-C10	70	4.36	116	11.8	0.603	1.4	12.54	976
	SF-0.5-C10	88	5.2	145	13.2	0.606	1.4	14.05	1134
	SF-1-C10	104	7.39	166	15.8	0.626	1.64	13.97	1868
D	NC-C15	45	5.23	106	10.9	0.424	1.64	9.89	850
	SF-0.5-C15	77	6.2	137	12.1	0.562	1.53	13.58	1047
	SF-1-C15	78	5.37	155	11.3	0.503	1.35	15.27	966

## Data Availability

The data is available upon request.
